# Improving Access to, Use of, and Outcomes from Public Health Programs: The Importance of Building and Maintaining Trust with Patients/Clients

**DOI:** 10.3389/fpubh.2017.00022

**Published:** 2017-03-08

**Authors:** Paul Russell Ward

**Affiliations:** ^1^Discipline of Public Health, Flinders University, Adelaide, SA, Australia

**Keywords:** trust, trustworthiness, public health practice, public health policy, sociological theory, cancer screening, childhood immunizations

## Abstract

The central argument in this paper is that “public trust” is critical for developing and maintaining the health and wellbeing of individuals, communities, and societies. I argue that public health practitioners and policy makers need to take “public trust” seriously if they intend to improve both the public’s health and the engagement between members of the public and public health systems. Public health practitioners implement a range of services and interventions aimed at improving health but implicit a requirement for individuals to trust the practitioners and the services/interventions, before they engage with them. I then go on to provide an overview of the theory of trust within sociology and show why it is important to understand this theory in order to promote trust in public health services. I then draw on literature in three classic areas of public health—hospitals, cancer screening, and childhood immunization—to show why trust is vital in terms of understanding and potentially improving uptake of services. The case studies in this paper reveal that public health practitioners need to understand the centrality of building and maintaining trusting relationships with patients/clients because people who distrust public health services are less likely to use them, less likely to follow advice or recommendations, and more likely to have poorer health outcomes.

## Centrality of Trust in Contemporary Society

Both theoretical and empirical literatures point to the idea that contemporary society is bound up with decreasing levels of trust (1, 2). This declining trust has been witnessed in healthcare (3–9) alongwith other institutions (1, 2, 10). Some authors argue that public distrust has become the default position in recent times, often evidenced by increasing numbers and strength of belief in conspiracy theories and is the “cultural logic of modernity” (11). Declining public trust has been linked to increased questioning about and confidence in science and experts to “have the right answers,” and the proliferation of information in the internet and social media, “*When the life-world is colonized by medical insecurity, medicalized subjects come to suspect the messenger and the knowledge they bear*” (12) (p. 524). When this public questioning and distrust is applied to public health, one may argue that public health and medical practitioners are no longer able to be regarded as the “experts” around health and illness, since “*all knowledge is tentative, corrigible and therefore open to subsequent revision or abandonment*…*Systems of expertise come to represent multiple sources of authority that are frequently contested and divergent in their implications*” (13) (p. 262). With the changing power base of medicine in society and the multiple sources of seemingly credible information about how people can/should manage their health and illness, understandably people begin to question who and what to trust. In addition, the increasing confidence and “rights” understood by patients and the public that they can, and should, challenge normative assumptions, such as all healthcare is equal, in their best interests, and trustworthy.

In increasingly complex and ever-changing times, sociologists argue that the public look toward so-called *expert systems* (e.g., public health, science, and politics) to make predictions about the future, thereby allowing the public to make decisions on the basis of trustworthy information provided by these expert system (14). However, Luhmann identifies the difficulties for expert systems to predict the future, “*To show trust is to anticipate the future. It is to behave as though the future were certain*” (15) (p. 10). However, the public health system cannot adequately predict future health needs, compelling/driving people to look for other sources of information and to question their trust in public health services (16) and also the systems which are perceived to support them. Indeed, Luhmann stated that “*one should expect trust to be increasingly in demand as a means of enduring the complexities of the future which technology will generate*” (15) (p. 16). The fact that we can log onto an internet search engine and obtain multiple, contested, and changing answers to our searches (e.g., what are the risks and benefits of childhood immunizations?) has led to a state of existential anxiety [“*no man’s land*” (17); in the “*gray zone*” (18); “*betwixt and between health*” (12)], which means that the public may question the validity of public health knowledge (*vis a vis* other information provided on the internet) and hence, the “trustfulness” of both public health practitioners and their system of knowledge. Indeed, Crawford stylishly suggests that “*People are left wondering about the efficacy of medical advice: as the map of danger is filled in, safe passage appears all the more difficult; but as the map of safe passage becomes illegible, people do not know what to believe or how to act in order to be safe*” (12) (p. 511). In other words, we cannot simply assume or expect that the public will trust the public health system, “*it has continually to be* ‘*won*’” (14) and “*and is therefore being constantly renegotiated with lay audiences*” (19). I use three case studies later in the paper to highlight these issues of trust in different aspects of the public health system.

## Conceptualizations of Trust

Trust has been comprehensively researched and theorized elsewhere (4, 20–27), although I will broadly cover the key points within the theoretical literature in order to highlight both “what trust is” and “why it is important” for public health practitioners and policy makers to think about it carefully when planning public health services, programs, and interventions.

Sociologists identify two types of trust: institutional and interpersonal. Interpersonal trust is regarded as an outcome of interpersonal interactions that people can learn in order to make decisions about future interactions (an individual uses past experiences of similar interactions to predict whether or not to trust someone in the future) (15, 26, 28, 29). I use the initial definition by Sabel (1993:1133) for interpersonal trust, “the mutual confidence that no party will exploit another’s vulnerability” (30), within this paper. However, this definition does not include the important addition of “power” within interpersonal relationships, so I extend the previous definition recognizing that to trust others, is to “accept the risks associated with the type and depth of the interdependence inherent in a given relationship” (31). For example, to place trust in a doctor or surgeon has potentially greater risks than placing trust in a shopkeeper or waiter. Institutional trust relates to people investing trust in a system or institution (as distinct from a person), such as a hospital, a medical clinic, or a cancer screening program. Hudson (32) argues that institutional trust is different from interpersonal trust. Mishler and Rose define institutional trust as “*the expected utility of institutions performing satisfactorily*” (33) (p. 31). Giddens argues that there are social/cultural norms underpinning the decision to trust (outside of actual experience), often based on a stylized idea of the institution (34). Indeed, Fukuyama (35) argues that “*trust arises when a community shares a set of moral values in such a way as to create expectations of regular and honest behavior*” (p. 153). My own research on trust in different public health services found that it is possible to have trust in one level but not necessarily the other. For example, in a study of trust in hospitals, patients in public hospitals trusted the individual doctors but not necessarily the government funding the hospitals (36). In a study of trust in colorectal cancer (CRC) screening, some cultural groups trusted and some distrusted the government funding the screening, and some groups trusted and others distrusted the health-care professionals involved in the screening (9, 10).

Sociologists argue that trust only exists when there is a deficit in knowledge by the person needing to trust (e.g., the patient, client), since there would be no need for trust in a situation where they had complete knowledge (37)—a decision in full knowledge would not require trust. In order for an individual to have “trust” in either a public health practitioner or institution, their decision is a combination of “good reason” (i.e., past experience of trusting relationships and good outcomes) a “leap of faith” that hopefully plugs the gap in their “partial understanding” (37). In this way, trust is a pledge, under conditions of uncertainty, to more than simply cognitive understanding (37). The smaller the “good reason” and the larger the “leap of faith,” the higher the risk of trusting. This is often the case in public health, when we cannot always predict whether a treatment will be effective, whether a screening test will be completely accurate, or whether public health practitioners will always act in the best interest of patients or the public.

When mistrust occurs, it often starts from interactions with the people who represent the systems or institutions (e.g., immunization nurses who represent immunization programs, or doctors who represent cancer screening programs). Giddens uses the term “access point” to identify the social situations in which the individual (e.g., public health practitioner) is perceived as representing a particular institution or social system, arguing that “*Although everyone is aware that the real repository of trust is in the abstract system, rather than the individuals who in specific contexts* ‘*represent*’ *it, access points carry a reminder that it is the flesh-and-blood people (who are potentially fallible) who are its operators*” (28) (p. 85). Using this logic, it would follow that institutional trust is built on, and predetermined by, interpersonal trust (38). This is critical for public health, since the public health practitioners therefore have a central role in developing, maintaining, and potentially rebuilding trust in public health systems and services.

## The Interplay Between Trust and Public Health

This section addresses the importance of trust in key areas of the social determinants of health: socioeconomic security, social inclusion, and social empowerment (39, 40).

The definition of socioeconomic security is based on the degree to which people have access to a range of services and systems, including good healthcare and education, safe housing, and appropriate employment opportunities. This domain has great historical credence in public health, and there is a plethora of public health policy (41–45) and research (46–50) in this area. Much of the sociological literature on trust in public health has focused on how trust impacts on patient access to and use of healthcare and highlights the significance of both interpersonal and institutional trust for developing socioeconomic security. In addition, there is a large amount of public health research highlighting the relationship between negative health outcomes and perceptions of economic and psychological insecurity and relative deprivation (51, 52). Therefore, there seems to be a relationship between trust and perceptions of socioeconomic security, fitting in with much of the research on social gradients in health (46, 47, 51, 52).

Social inclusion is defined by the extent to which people have appropriate access to services and systems that they require for normal daily living (e.g., health, education, and welfare), and also feel integrated or included within the social relations of “everyday life” (39, 40). Social inclusion focuses on whether people “feel part of” or “included in” society, which is inextricably linked to both interpersonal and institutional trust (or distrust) (2, 22, 53–55).

In terms of the relationship between trust and social inclusion, my view is that people and groups cannot feel and be completely “included” unless there are trusting relations, which need to be reciprocated by both parties in the relationship and also trusted portals of access. These trusting relations may be in terms of more micro-level processes—an individual who has recently moved to a new country getting access to and feeling included in local networks. These may also be on a more macro level—policy makers and/or practitioners may exclude (consciously or sub-consciously) particular marginalized groups because they are perceived to be untrustworthy. My own research on both interpersonal and institutional trust across six different Asia-Pacific countries highlights the relationship between social inclusion and trust (1, 2, 39). In these studies, population groups with higher levels of perceived social inclusion also had higher levels of both interpersonal and institutional trust.

Social empowerment is defined by the extent to which an individual’s personal capabilities are enabled or disabled within society (40). Social empowerment builds on both social inclusion and socioeconomic security by exploring the enabling (or disabling) factors (e.g., human rights) which empower people to act as autonomous humans. Some research has shown that in situations where individuals exhibit generalized levels of distrust, they also feel completely disempowered—they feel cut off from and let down by various sources of power and therefore that they do not have a “voice” to enable situations to change for the better (56). Of course, the relationship between distrust and disempowerment can work both ways, with both negative feelings feeding off each other.

## Key Case Studies in of Trust in Public Health

I outline literature and some of my own research on trust in different areas of the public health system: hospitals, cancer screening, and childhood immunizations. These have been chosen because they serve different functions, involve different types of health-care practitioners, and have different treatment/prevention modalities, yet they all suffer from questioning of trust by groups of patients/clients.

## Trust in Hospitals

A number of public health services and programs are provided in hospitals and dealing with the issue of trust or distrust in hospitals is increasingly important, given the declining trust in Western health-care systems (4, 57). There is a need for more research on trust in hospitals and health-care systems more broadly (58), with the authors of one paper stating that we need to “*understand, protect and restore public trust in the health care system*” (58) (p. 1). If members of the public distrust hospitals and/or the health-care professionals working in them, it creates a difficult situation for them because distrust related to poorer patient outcomes. Low levels of trust are associated with increased risk of psychological distress (59), and patients with low levels of trust are more likely to be in low socioeconomic groups, less likely to seek or access healthcare, less likely to accept healthcare recommendations or maintain continuity of care, and more likely to avoid healthcare, including hospitals, entirely (60). Conversely, higher trust in healthcare enhances the likelihood of return for follow-up care, increases patient adherence to therapies, facilitates health information exchange, and enables providers to encourage necessary behavioral changes (61–65).

An Australian qualitative study found that patient’s trust in private hospitals was one of the key reasons why they chose to buy private health insurance (66), and an Australian survey found that private hospitals are invested with higher trust than public hospitals (67). In contrast, research in the US found that publically funded healthcare is invested with higher trust than privately funded healthcare (61). The higher trust in publically funded healthcare in the US may be due to public health-care systems having more transparency, public accountability, and a lack of profit-related motives, thereby increasing public trust in relation to privately funded healthcare that is regarded as being driven by profit-related motives (68).

My own research on trust in public and private hospitals in Australia found that public and private patients made very different assessments about trust in hospitals. Patients in private hospitals made trust-decisions in a very similar manner to consumers making purchases—they assessed the various options about possible doctors and/or hospitals, thought about which one was the most trustworthy and then made their choice. In this way, trust and choice went hand in hand. In making their trust-based choice, they relied on both objective and subjective assessments of the reputation of the doctor and/or the hospital, linking better reputation to higher quality healthcare. Patients with private health insurance could “shop around” for particular hospitals and particular doctors in whom they either had positive previous experience or friends/family had similar positive experience. As noted earlier in the section on the sociology of trust, this prior experience reduces the risk in placing trust (reduces the “leap of faith”) because the decision to trust is based on a more “reasoned decision”. Conversely, patients in public hospitals had no choice in their hospital and/or doctor, since they had been referred (often from their general practitioner) and saw whichever doctor was on duty at that time. The patients in public hospitals often did not know their doctor (larger “leap of faith”) and in order to place their trust, patients stated that doctors in public hospitals would try to “do their best”, thereby being trustworthy. This level of trust goes back to the definition of trust which is about trusting someone because you think they will do their best for you and assuming that they will not try to do harm. Our data show that both public and private patients can have “trust” in their hospital doctors, but they are based on different rationales and types of evidence. The private patients exhibited a kind of “active trust,” and the public patients exhibited a kind of “resigned trust”.

## Trust in Cancer Screening

There is a great deal of research on trust in various forms of cancer screening, but I will focus specifically on CRC screening here. There are a number of known barriers to people undertaking CRC screening, including a lack of knowledge about both their personal CRC risk factors and knowledge about screening (69–71), lack of trust (72, 73), fear of the screening test (74), and broader concerns about the effectiveness and purpose of cancer screening (73).

Making a decision about whether or not to participate in a population-based screening program such as CRC screening is likely to involve a trust-based decision because the majority of potential participants will be asymptomatic and are likely to have low knowledge about the program or indeed CRC, therefore requiring trust *via* a “leap of faith” (20). In other words, most members of the public do not have in-depth knowledge of screening programs, and therefore rely on “trusting” particular public health practitioners or public health institutions—this “trust” allows them to make decisions and allow for their imperfect knowledge (75).

Most of the research on trust in cancer screening programs has understandably focused on screening methods that includes the intervention of medical professionals (72, 76, 77), since the researchers can examine the impact of interpersonal trust in improving screening rates. Indeed, people with higher trust in their doctor are more likely to undertake screening tests (72, 77, 78). US research with women found higher trust in screening programs undertaken within health centers (e.g., Pap smear, clinical breast exam) but lower trust in screening programs requiring women to perform the tests themselves and at home (e.g., breast self-examination and CRC screening) (77). The higher trust in screening undertaken in health centers as opposed to more “faceless” screening at home maybe due to the involvement of interpersonal (e.g., doctor) trust—this fits in with the idea of the “access point” between interpersonal and institutional trust mentioned earlier in the paper. However, a number of population-based CRC screening programs, including Australia, do not involve any health-care professionals in the initial screening test, which involves people performing an immunochemical fecal occult blood test in their own home and then mailing it to a central laboratory.

My own, along with colleagues, research on trust in CRC screening in Australia identified different types of trust and distrust by different population groups (9, 10). We highlighted the nuances and complexities involved in the trustworthiness of the CRC screening program, which included trust considerations at different levels: interpersonal relationships with people perceived as linked to the CRC screening program (e.g., GP, Aboriginal Health Worker), local area issues that impacted on the program (e.g., trustworthiness of postal system or local health center) and national political issues (e.g., trustworthiness of the government and particular politicians seen the “represent” the government). At a more abstract level, there was questioning about the ability of doctors to actually diagnose or treat cancer (questioning the “point” of screening) and the scientific procedures in laboratories to identify blood in the small amount of feces required for the screening test (questioning the validity of the screening). Levels of trust differed between cultural groups, with the Indigenous participants having mistrust in government and services run by the government, including health services, the postal service (required to obtain and send the screening test) and the CRC screening program. This mistrust in government led Indigenous participants to be much less likely to take part in the CRC screening program. In contract, Anglo-Australian and Iranian groups had much higher trust in the government, leading them to be more likely to undertake the CRC screening. In order to improve CRC screening (and probably engagement with many more public health services), the issues of broad mistrust in government need to be addressed in order to increase trustworthiness of, and trust/participation in public health services, particularly for Indigenous Australian people.

## Trust in Childhood Immunizations

Childhood immunization programs have been so effective in the elimination of infectious disease that they have become a victim of their own success (79), with some people now questioning the need for childhood immunizations due to their perception that certain diseases are rare and therefore less concerning (80). Public health practitioners thus have to engage with, and promote the benefits of, vaccination to groups who are increasingly unlikely to have encountered some of the diseases they are being asked to vaccinate their children against.

The increasing debate in Western society regarding the real or perceived adverse events following vaccination has made some parents “uneasy” about the decision to vaccinate their children (81). This “unease” or “uncertainty” is called “vaccine hesitancy” (82), and approximately 20–30% of all parents in some countries are vaccine hesitant (80, 83). The literature attempting to understand this phenomenon reveals mistrust as a key factor, but there lacks a rich theoretical exploration of the interaction between trust and vaccine hesitancy and specifically how trust in vaccines is eroded and maintained.

There are a number of concerns that parents hold regarding vaccines, mostly centered on concerns about vaccine safety (84). The immunization process induces complex, emotional decisions in some parents who are faced with potentially difficult choices, such as attempting to balance the individual rights of their child with the broader health protection of the community (85). Other widely held concerns by vaccine hesitant parents are as follows: the perceived high number of vaccinations given to children; that health professionals may provide inadequate information; that health professionals are perceived to be unwilling to spend adequate time providing vaccine information; and that vaccines may be perceived to overload their child’s immune system, vaccine components may be harmful, and alternative medicines may suffice in place of vaccines (84). The final concern regarding vaccine hesitancy concerns trust. Not only do some parents distrust the medical system but anything recommended by government institutions (83). A core research question that resulted from the 2014 report by American Academy of Arts and Sciences, entitled “*Public Trust in Vaccines: Defining a Research Agenda*” was, “To what extent does vaccine hesitancy result from broader distrust in government and science” [(83) p. 10]. This question resonates with other recent literature which cites “trust” as critically important in the decision for parents to vaccinate (86–89). Trust in vaccines and vaccination is complex: it describes a continuum of trust from the funding of immunology research, to vaccine design and manufacture, through government decision making regarding which vaccinations to fund for immunization programs, to the point at which a vaccine is administered by the medical provider to the individual. The parental decision to vaccinate or not is both the beginning and the end point of the vaccine journey and if *dis*trust is evident at any point in this journey then there is a potential for vaccine rejection.

Public trust in vaccinations, and the health professionals who promote them, has been identified in the literature as pivotal in determining whether parents will decide to immunize their child (80, 90). Parental perceptions of insufficient, biased, poorly communicated advice from health-care providers is noted in the literature as key to a lack of trust in vaccinations (90) with the result that individuals may turn to the internet for advice, where they may compound their confusion with a multitude of conflicting and unregulated material so that it is difficult to discriminate between the evidence-based sources and those based on anecdote and misinformation (87, 91).

Maintenance of institutional trust is paramount to immunization programs. For example, concerns regarding trust in institutions involved in vaccinations during the 2009 influenza H1N1 pandemic led to increasing hesitancy to vaccinate, linked to conspiracy theories, and speculation that the pandemic response was influenced by commercial interests (79). This distrust was further promulgated in Australia when the 2010 seasonal influenza vaccine for children was withdrawn due to an observed increase in febrile convulsions, later found to be linked to one vaccine brand. Despite the resumption of the vaccine program with other vaccine brands, persistent mistrust, and confusion is linked to a decline in influenza vaccination coverage. It is also argued that institutional trust is being eroded by current social trends toward patient advocacy, empowerment, and patient choice, being at odds with the traditional approach to public health programs, which is increased further with virtually unlimited access to health information *via* sources, such as social media and the internet (79). Given the importance of understanding parental (dis)trust in childhood immunizations, I am currently part of a research team undertaking in-depth qualitative research to further develop our understanding. Our first paper from this study outlines the ways in which broad distrust in multinational pharmaceutical companies impacts some parents trust in childhood vaccinations and their decisions not to vaccinate their children (92). A number of parents perceived that pharmaceutical companies were motivated purely by profits and had the global power and reach to influence governments and research institutions and thus questioned whether they were indeed “working for the best interests of children”, a key issue in trustworthiness. The immunizations were therefore imbued with distrust, not necessarily due to the ingredients of the vial, but the various institutions that have created and marketed it. Rebuilding trust in this example may require “distancing” the immunization from the pharmaceutical companies and being clearer on the independence of researchers (and the scientific system) and governments (and the political system) in making decisions on childhood immunization policy and practice.

## Conclusion

Contemporary public health systems are located historically and culturally within a society whereby individuals question, research, interrogate, and seek alternatives to “traditional” approaches to health and illness. The push to modernity has meant that public health practitioners can no longer just assume that patients or the public will simply “trust” them because of their position in society or their extensive training. Therefore, trust needs to be won and kept because “trust comes on foot and goes away of horseback” (93) (p. 389). In other words, once trust has been lost, it is very difficult to regain it. This is critically important because, as I have shown using numerous examples from different areas of public health, people who distrust public health services are less likely to use them, less likely to follow advice or recommendations, and more likely to have poorer health outcomes. Therefore, public health practitioners need to understand the centrality of trust in their roles. They need to understand the importance of engaging meaningfully and in a trustworthy fashion to build and maintain trust in those groups who are currently mistrusting and to maintain trust in all other groups.

## Author Contributions

PW developed the ideas, reviewed the literature, and wrote the paper.

## Conflict of Interest Statement

The author declares that the research was conducted in the absence of any commercial or financial relationships that could be construed as a potential conflict of interest.
